# GNL3 Regulates SIRT1 Transcription and Promotes Hepatocellular Carcinoma Stem Cell-Like Features and Metastasis

**DOI:** 10.1155/2022/1555670

**Published:** 2022-04-07

**Authors:** Songyan Zhang, Haoran Zhao, Yang Chen, Yubao Zhang

**Affiliations:** Department of Hepatobiliary and Pancreatic Surgery, Affiliated Cancer Hospital of Harbin Medical University, Harbin, Heilongjiang 150040, China

## Abstract

The expression of GNL3 in hepatocellular carcinoma was detected, and its effect on the proliferation and metastasis of hepatocellular carcinoma cells was investigated. Hepatocellular carcinoma and adjacent tissues were collected. The mRNA and protein expression levels of GNL3 were detected by qRT-PCR, Western blot, and immunohistochemistry. The relationship between GNL3 and the prognosis of liver cancer was analysed using public databases. A GNL3 interfering plasmid was constructed, and the effects of GNL3 on the proliferation of HepG2 and PLC-PRF-5 hepatoma cells were detected by the CCK-8 method. Transwell chamber assays were used to detect the effects of GNL3 on the migration and invasion of hepatocellular carcinoma cells. The effects of GNL3 on SIRT1 expression and stem cell markers were analysed. The effect of GNL3 on the proliferation of hepatocellular carcinoma was detected in a subcutaneous tumor-bearing animal model. The results showed that the mRNA and protein levels of GNL3 were higher than those of adjacent tissues. The overall survival (OS) of HCC patients with high GNL3 expression was worse. In vivo and in vitro experiments confirmed that silencing GNL3 could inhibit the proliferation, migration, and invasion of hepatocellular carcinoma cells. Mechanistic studies have shown that GNL3 regulates SIRT1 expression. GNL3 mediates the stem cell-like properties of HCC cells through SIRT1. In conclusion, this study found that GNL3 increased expression in hepatocellular carcinoma, which promoted the malignant biological behavior of hepatocellular carcinoma cells and was related to the cell dry phenotype. This study has certain significance in evaluating the prognosis of HCC patients.

## 1. Introduction

Hepatocellular carcinoma (HCC) is one of the most common malignant tumors in clinical practice [1, 2]. Studies have shown that the most important factors affecting the survival of HCC patients are postoperative recurrence and distant metastasis [3]. Therefore, determining the risk factors related to HCC recurrence and metastasis and exploring its cancer-promoting mechanism will contribute to the formulation of clinical diagnosis and treatment strategies and the development of antitumor drugs [4].

HCC is more heterogeneous than other solid organ tumors. The tissue interior is composed of a variety of subclonal tumor cells [5]. Due to gene mutation and recombination, different subclonal tumor cell lines have significantly different tumor genomes [6]. It has different functions, including stem cell characteristics and chemotherapy resistance. Tumor stem cells significantly enhanced the viability of HCC cells [7]. Therefore, detection of the regulatory mechanism of HCC cell stem cell-like cells plays an important role in revealing the molecular mechanism of HCC occurrence and development [8]. Tumor stem cells are a small group of specific tumor cells. It is considered to be a major cause of tumorigenesis, drug resistance, and recurrence [9]. GNL3 (guanine nucleotide-binding protein like 3) is a member of the MMR1/HSRICTP binding protein family [10]. GNL3 plays an important role in stem cell proliferation [11, 12]. At present, the function and mechanism of GNL3 in HCC stem cells need further study.

Silent information regulator 1 (SIRT1) is a newly discovered class of histone deacetylases dependent on nicotinic amine adenine dinucleotides [13]. SIRT1 is expressed in a variety of mature tissues and is abundant in early embryos, i.e., germ cells [14, 15]. SIRT1 is a key link involved in normal cell physiological activities and is closely related to a variety of human tumors [16]. In HCC, SIRT1 is the only member of the sirtuin family with sustained high expression. It plays a very important role in maintaining the occurrence of liver cancer [17] and affects the proliferation, invasion, and migration of HCC to a certain extent. In addition, SIRT1 also has a certain influence on chemotherapy resistance in liver cancer. The increased expression of SIRT1 is correlated with tumor treatment and prognosis [18]. However, the relationship between GNL3 and SIRT1, especially whether GNL3 can regulate the expression of SIRT1, has not been reported.

As a promoter of cancer, GNL3 regulates the expression of a large number of genes and plays an important role in tumors. However, to date, the expression of GNL3 in HCC and whether SIRT1 is involved in its regulation remains unclear. This study aimed to observe the expression of GNL3 in human HCC tissues and its correlation with the prognosis of HCC patients. On this basis, RNA interference technology was used to knock out GNL3 and analyse the growth and metastasis of HCC cell lines. This study further analysed the stem cell-like properties and mechanism of GNL3 through SIRT1 in hepatocellular carcinoma cells.

## 2. Methods

### 2.1. Liver Cancer Tissue Collection

Case samples were collected from 20 patients with primary hepatocellular carcinoma who underwent surgical resection and were pathologically confirmed in our hospital from March 2021 to October 2021. The pathological type of liver cancer collected was hepatocellular carcinoma, which was independently evaluated by 2 pathologists. The diagnosis was based on the Chinese Society of Clinical Oncology (CSCO) Guidelines for the Diagnosis and Treatment of Primary Liver Cancer in 2020. None of the patients received any other treatment before surgery. This study was approved by the Ethics Committee of the Affiliated Cancer Hospital of Harbin Medical University.

### 2.2. Bioinformatics Analysis

The Human Protein Atlas database (https://www.proteinatlas.org/) was used to analyse GNL3 RNASeq data from cancer patients. The Kaplan–Meier method was used to calculate the overall survival of HCC patients with low or high GNL3 expression.

### 2.3. Immunohistochemical Assay

Tissue sections of liver cancer specimens were baked for 2 h in a 65°C oven. After conventional dewaxing of xylene and hydration with gradient ethanol, the sections were rinsed with PBS 3 times (5 min/wash). The effects of endogenous peroxidase were removed by incubation with 3% H_2_O_2_ at room temperature for 10 min. The tissue sections were rinsed with PBS 3 times (5 min/wash) and completely immersed in a citric acid solution at pH 6.0. High-pressure repair was performed for 3 min, followed by cooling at room temperature. PBS was rinsed 3 times (5 min/wash), and then GNL3 (Abcam, USA, 1 : 100) and Ki67 (Abcam, USA, 1 : 100) primary antibody working solutions were added overnight at 4°C. Rewarming for 40 min to prevent strip removal and washing 3 times with PBS (5 min/wash). The second antibody working solution was dropped and incubated at room temperature for 1.5 h. All the following: DAB color rendering, hematoxylin redyeing, dehydration, transparency, and sealing were carried out. Microscopic observation, grading, and drawing were also performed.

### 2.4. Cell Culture and Transfection

Hepatocellular carcinoma cells were purchased from ACTT. Cell lines were cultured in DMEM high glucose medium containing 10% fetal bovine serum (reagents and serum were from Gibco). Culture was carried out at 37°C in a relatively saturated incubator with 5% CO_2_ (Thermo Company, USA). The experimental cells were in a logarithmic growth phase. Cells at the logarithmic growth stage were selected to prepare a single-cell suspension at a density of 10 × 106/mL. It was then inoculated into 6-well culture plates. After 24 h of culture, the cells grew to 80% confluence and were transfected when fusion was performed. According to the Lipo2000 kit instructions, plasmids pcDNA3.1-NC, pcDNA3.1-GNL3, shRNA, shGNL3, and shSIRT1 were transfected. Fresh medium containing fetal bovine serum was replaced 6 h later. Transfection efficiency was detected after 48 h of culture.

### 2.5. Western Blot

The abovementioned tissues and cells were lysed with RIPA reagent (containing protease and phosphatase inhibitors). Protein concentration was determined by the BCA method. Whole-cell lysates with the same amount of protein (30 *μ*g) were subjected to SDS-PAGE. The isolated protein was electrically transferred to a PVDF membrane. The immunoblotting membranes were sealed in TBST buffer containing 5% skim milk powder at room temperature for 1 h, and rabbit anti-human GNL3, Nanog, ALDH1, Oct4, and GAPDH antibodies were added (Abcam, USA, 1 : 5000 dilution). GAPDH monoclonal antibody (Abcam, USA, 1 : 7500 dilutions, as internal reference) was incubated overnight at 4°C. The TBST membrane was washed 3 times for 15 min each, and HRP-labeled anti-rabbit secondary antibody (Abcam, USA, 1 : 15000 dilution) was dropped. The membrane was incubated at room temperature for 1 h, and the membrane was washed 3 times with TBST. ECL chemiluminescence detection (American Thermo Company). The experiment was repeated three times.

### 2.6. Transwell Assay

Transwell cells were placed in a new 24-well plate, and 100 *μ*l of Matrigel without FBS dilution was added to the upper chamber. Place in an incubator at 37°C for 1 h. Cells were added to each chamber, and 600 *μ*l of medium containing 10% FBS was added to the lower chamber. Incubated at 37°C for 48 h with 5% CO_2_. The nonmetastatic cells in the chamber were gently removed with a cotton swab, and the chamber was fixed in 4% paraformaldehyde for 30 min. One to two drops of staining solution were dropped onto the lower surface of the membrane to stain and transfer cells for 1∼3 min. Soak and rinse the chamber several times, and air dry. The number of metastatic cells in each group was calculated.

### 2.7. CCK-8 Assay

When the confluence of cell growth reached approximately 70%, the cells were digested with trypsin and prepared into a single cell suspension (2.5 × 10^5^ cells/mL). Inoculate 100 *μ*L of cell suspension into a 96-well plate. Ten microliters of CCK-8 solution (10 mg/ml) and 100 *μ*L of culture solution were added to each well, and the cells were cultured in an incubator for 2 h. The optical density at 450 nm (*D*) was measured with a microplate reader, and the proliferation inhibition rate was calculated ((*D* control group–*D* experimental group) (/*D* control group − *D* blank group) × 100%).

### 2.8. Plate Clone Formation Assay

Cells from each group were inoculated into 6-well plates (10 × 10^3^ cells/well). After culturing in the incubator for 14 days, the culture medium was changed every 3 days. Cell status was observed, and the culture was terminated when the number of colonies was >50. The culture medium was discarded, fixed with formaldehyde for 30 min, and washed with PBS. The number of clones was counted by staining with 500 *μ*L of Gisam solution.

### 2.9. qRT-PCR

Total RNA was extracted from the cells and reverse transcribed. Upstream PCR detection was performed on a fluorescence quantitative PCR instrument (TP800, TAKARA Company, Japan). The reaction conditions were as follows: predenaturation at 95°C for 15 seconds, then denaturation at 95°C for 5 seconds, and annealing and extension at 60°C for 30 seconds. A total of 45 cycles were performed, and the absorbance value was read each time during the extension phase. The housekeeping gene GAPDH (glyceraldehyde-3-phosphate dehydrogenase) was used as the internal reference gene. The primer sequences were as follows: upstream 5′-TGACTTTCAA-CAGCGACACCCA-3′; downstream 5′-CACCCTTTTGCTTG-TAGCCAAA-3′. The 2^−ΔΔCT^ method was used to analyse the expression levels of genes in each cell line relative to the internal reference genes. If the expression level of the GNL3 gene in liver cancer tissue is higher than that in the corresponding adjacent tissues, the expression of GNL3 is upregulated.

### 2.10. Immunofluorescence Staining

The cell slides were fixed in 4% paraformaldehyde for 10 min. 0.25% Triton to permeabilize for 5 min. 5% BSA was sealed for 30 min. GNL3 and SIRT1 primary antibodies (Abcam, USA, 1 : 50) were added and incubated overnight at 4°C. Rinse 3 times with PBS for 5 min/time. Clonal fluorescent antibody (Jiangsu Biyuntian Biotechnology Co., Ltd., 1 : 100) was added and incubated for 1.5 h. Fluorescence microscopy was used to observe and photograph.

### 2.11. Subcutaneous Graft Tumor Model

Fourteen specific pathogen-free (SPF) female BALB/c nude mice were aged 6 to 8 weeks and weighed 16 to 20 g. 7 in each group. HepG2 tumor cell suspension (0.2 mL) was subcutaneously inoculated into the right dorsal shoulder of nude mice. The mental state, diet, defecation, activity, and tumorigenesis of nude mice were observed daily after inoculation. After tumor formation, the long diameter (*L*) and short diameter (*W*) of the transplanted tumor were measured and recorded every 2 days with vernier calipers. Graft volume = (*L* × *W*^2^)/2. The nude mice in each group were euthanized on day 35. The subcutaneous graft tumor was removed. The liver and some tumor tissues were fixed in a 10% paraformaldehyde solution, and then paraffin sections were prepared for later use. The metastases were detected by HE staining.

### 2.12. Statistical Analysis

The experimental data were collated and analysed by GraphPad Prism 5.0 statistical software. A paired *t*-test was used to compare the differences between paired measurement data. The independent sample *T*-test was used to compare the two groups of independent measurement data. Survival analysis was performed by the Keplan–Meier method. Comparisons between multiple groups were analysed using one-way ANOVA. *P* < 0.05 was considered statistically significant.

## 3. Results

### 3.1. Expression of GNL3 in Liver Cancer Tissue Samples and Its Relationship with the Prognosis of Liver Cancer Patients

**Figure 1:**
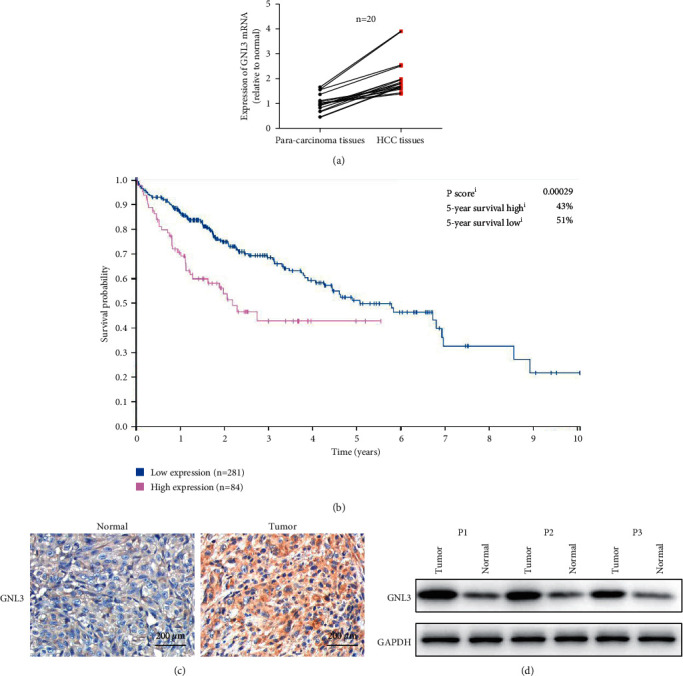
The expression of GNL3 in liver cancer tissue samples and its relationship with the prognosis of liver cancer patients. (a) Collected liver cancer tumor tissue and adjacent nontumor tissue samples were matched from 20 liver cancer patients. The qRT-PCR method was used to detect the level of GNL3 mRNA in tissue samples (*n* = 20). (b) The Kaplan–Meier method was used to calculate the overall survival of liver cancer patients with low or high GNL3 expression. (c) Immunohistochemical method to detect the expression of GNL3 in liver cancer and adjacent nontumor tissues. (d) Representative blot showing the expression of GNL3 protein in four pairs of tissue samples.

Real-time fluorescence quantitative PCR detection results showed that the average expression level of the GNL3 gene in liver cancer tissues was higher than that in adjacent tissues of 20 patient specimens (Figure 1(a))). The prognosis of patients with high GNL3 expression (*n* = 84) was significantly worse than that of patients with low GNL3 expression (*n* = 281) (Figure 1(b)). Immunohistochemical results showed that GNL3 was highly expressed in HCC tissues (Figure 1(c)). Western blotting was used to detect GNL3 protein expression in two pairs of tissue samples. The results showed that the expression level of the GNL3 gene in liver cancer tissues was higher than that in adjacent tissues (Figure 1(d)).

### 3.2. Effects of GNL3 on the Growth and Migration of Hepatocellular Carcinoma Cells

**Figure 2:**
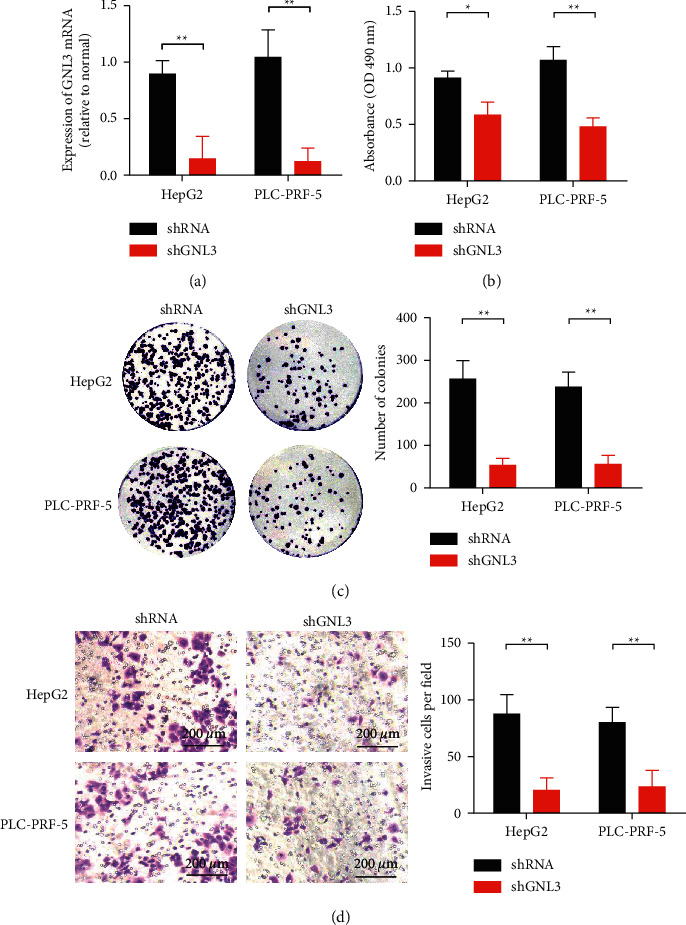
The effect of GNL3 on the growth and migration of liver cancer cells. (a) qRT-PCR was used to detect transfection efficiency 48 h after transfection. (b) CCK-8 assay to evaluate cell viability after transfection. (c) Colony formation test to detect the clone formation ability of HepG2 and PLC-PRF-5 cells. The transfected liver cancer cells were cultured in medium containing 10% fetal bovine serum for 2 weeks. (d) The transwell method to evaluate the invasion ability of HepG2 and PLC-PRF-5 cells. ^*∗*^*P* < 0.05and ^*∗∗*^*P* < 0.01.

qRT-PCR was used to detect the transfection efficiency 48 h after transfection (Figure 2(a)), and the results showed that shGNL3 could reduce the expression of GNL3 in HepG2 and PLC-PRF-5 HCC cells. In HepG2 and PLC5 cells, the inhibition rates of shRNA were 83% and 88%, respectively. CCK-8 detection results showed that the growth rate of HepG2 and PLC-PRF-5 HCC cells in the shGNL3 group was significantly slower than that in the NC group (Figure 2(b)). The results of the clonal formation experiment showed that the clonal formation rate of HepG2 and PLC-PRF-5 HCC cells in the shGNL3 group was significantly lower than that in the NC group (Figure 2(c)). Transwell results showed that the number of invading HepG2 and PLC-PRF-5 HCC cells in the shGNL3 group was significantly lower than that in the NC group (Figure 2(d)). These results suggest that GNL3 gene knockdown can significantly inhibit the proliferation and metastasis of HCC cells.

### 3.3. Effects of GNL3 on the Maintenance of Stem Cell-Like Properties of Hepatocellular Carcinoma Cells

**Figure 3:**
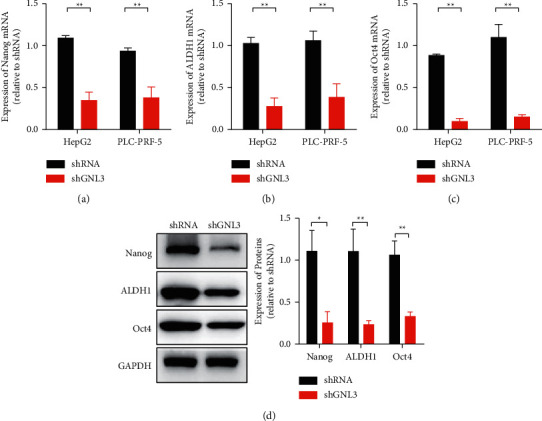
The effect of GNL3 on the maintenance of stem cell-like properties of liver cancer cells. (a) qRT-PCR to detect Nanog mRNA expression. (b) qRT-PCR to detect ALDH1 mRNA expression. (c) qRT-PCR to detect the expression of Oct4 mRNA. (d) Western blotting was used to detect the protein expression of Nanog, ALDH1, and Oct4. ^*∗*^*P* < 0.05and ^*∗∗*^*P* < 0.01.

qRT-PCR and western blotting were used to analyse the relationship between GNL3 and cell dry marker-related molecules. The stem cell markers Nanog, ALDH1, and Oct4 were selected for analysis. The results showed that the mRNA expression levels of Nanog, ALDH1, and Oct4 decreased after GNL3 silencing (Figures 3(a)–3(c)). The western blot results were consistent with the qRT-PCR results. After knocking down GNL3, the protein expression levels of Nanog, ALDH1, and Oct4 decreased (Figure 3(d)).

### 3.4. GNL3 Upregulated SIRT1 Expression

**Figure 4:**
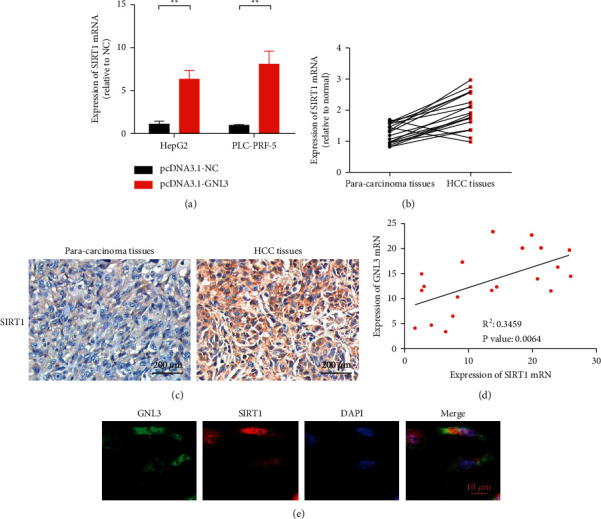
GNL3 upregulates the expression of SIRT1. (a) After overexpression of GNL3, the expression of SIRT1 in the transfected liver cancer cells was detected by qRT-PCR. (b) Collected liver cancer tumor tissue and adjacent nontumor tissue samples were matched from 20 liver cancer patients. The qRT-PCR method was used to detect the level of SIRT1 mRNA in tissue samples. (c) Immunohistochemical detection of SIRT1 expression in human liver cancer tumor tissue and adjacent nontumor tissue specimens. (d) Pearson's correlation coefficient was used to analyse the relationship between GNL3 and SIRT1. (e) Immunofluorescence to detect the colocalization of GNL3 and SIRT1. ^*∗∗*^*P* < 0.01.

The effect of GNL3 on SIRT1 was analysed by qRT-PCR. The results showed that overexpression of GNL3 upregulated SIRT1 expression (Figure 4(a)). The real-time fluorescence quantitative PCR detection results showed that in 20 patient samples, the average expression level of the SIRT1 gene in HCC tissues was higher than that in corresponding adjacent tissues (Figure 4(b)). Immunohistochemical results showed that SIRT1 was highly expressed in HCC tissues (Figure 4(c)). The relationship between GNL3 and SIRT1 was analysed by the Pearson correlation coefficient. The results showed that there was a significant positive correlation between GNL3 and SIRT1 expression levels (Figure 4(d)). Costaining results of immunofluorescence showed that GNL3 and SIRT1 were colocalized (Figure 4(e)).

### 3.5. GNL3 Mediates the Stem Cell-Like Properties of HCC Cells through SIRT1

**Figure 5:**
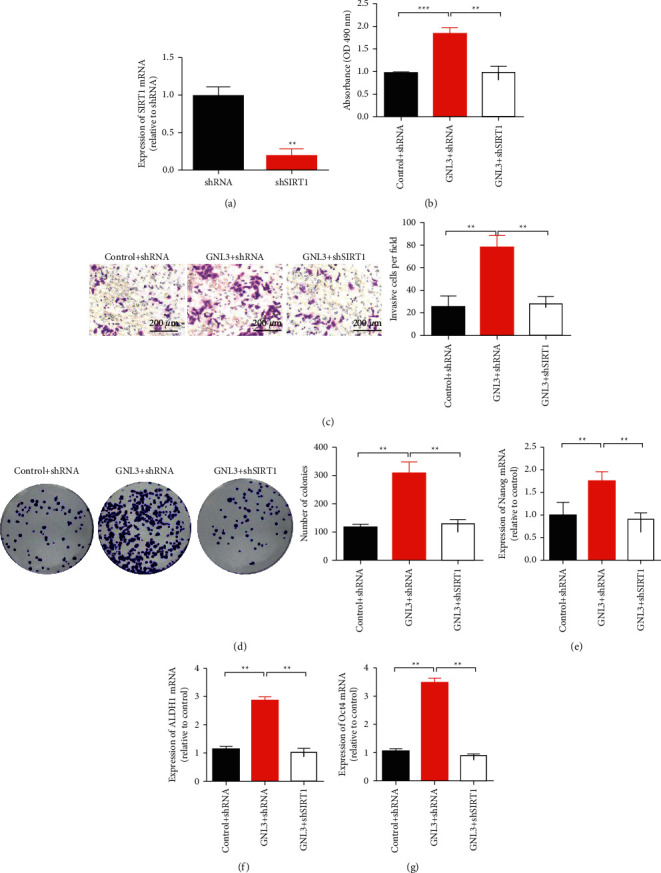
GNL3 mediates the stem cell-like properties of liver cancer cells through SIRT1. (a) qRT-PCR was used to detect transfection efficiency 48 h after transfection. (b) The CCK-8 method was used to evaluate cell viability after transfection. (c) Transwell assay to determine the invasion ability of liver cancer cells after transfection. (d) Evaluation of the colony forming ability of liver cancer cells after transfection by colony formation experiments. (e) qRT-PCR to detect Nanog mRNA expression. (f) qRT-PCR to detect ALDH1 mRNA expression. (g) qRT-PCR to detect the expression of Oct4 mRNA. ^*∗∗*^*P* < 0.01and ^*∗∗∗*^*P* < 0.001.

qRT-PCR was used to detect the transfection efficiency 48 h after transfection, and the results showed that shSIRT1 could reduce the expression level of SIRT1 in HCC cells (Figure 5(a)). In HepG2 cells, the inhibition rate of shRNA was 81%. CCK-8 assays showed that the growth rate of hepatoma cells in the GNL3 overexpression group was significantly higher than that in the NC group. Meanwhile, the cell proliferation rate decreased after simultaneous transfection of GNL3 + shSIRT1 (Figure 5(b)). Transwell and clonogenesis results showed that the invasion number and clonogenesis rate of HCC cells in the GNL3-overexpressing group were significantly higher than those in the NC group (Figures 5(c) and 5(d)). Meanwhile, GNL3 + shSIRT1-transfected GNL3 + shSIRT1 simultaneously reduced the number of invaded cells and the rate of clone formation. After overexpression of GNL3, the expression levels of Nanog, ALDH1, and Oct4 were upregulated. In contrast, the expression levels of Nanog, ALDH1, and Oct4 decreased after simultaneous transfection of GNL3 + shSIRT1 (Figures 5(e)–5(g).

### 3.6. Effect of GNL3 Gene Knockdown on Hepatocellular Carcinoma Tumor Growth and Liver Metastasis

**Figure 6:**
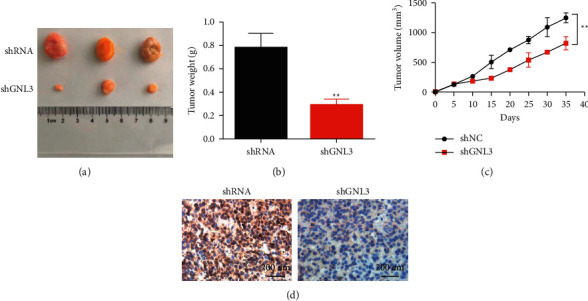
The effect of GNL3 gene knockdown on liver cancer tumor growth and liver metastasis. (a) Transfected liver cancer cells were injected subcutaneously into the flanks of BALB/c nude mice (*n* = 7 in each group) to establish a mouse liver cancer xenograft model. (b) At the end of the experiment, the statistical results of the tumor weight. (c) The tumor volume was recorded, and the growth curve was drawn. (d) Tumor tissue section and Ki-67 immunostaining (200 times magnification). ^*∗∗*^*P* < 0.01.

Transfected hepatocellular carcinoma cells were injected subcutaneously into BALB/C nude mice (*n* = 6 for each group) to establish a mouse hepatocellular carcinoma xenotransplantation model. The results showed that with the extension of time, the growth rate of transplanted tumor volume in nude mice of the shGNL3 group significantly slowed down, and the tumor weight also decreased (Figures 6(a)–6(c)). Immunohistochemical staining was used to detect the expression level of Ki-67 after different treatments. The results showed that the expression of Ki-67 decreased after knocking down GNL3 (Figure 6(d)).

## 4. Discussion

Liver cancer is the sixth most common malignant tumor in humans [19]. According to the latest global cancer statistics, there were 841,000 new cases and 782,000 deaths in 2018, which is a serious threat to human life and health [20]. Due to the high recurrence and metastasis rate of liver cancer and poor prognosis, in-depth research on the molecular mechanisms of liver cancer has always been the focus and difficulty in the field of liver cancer research [21, 22].

GNL3 is a member of the MMR1/HSR1CTP-binding protein family and is widely rich in nuclear proteins in stem cells [23, 24]. GNL3 plays a key role in early embryonic development [25]. Its biological significance has been clearly stated in a mouse animal model of embryonic death after GNL3 deletion [26, 27]. GNL3 is highly expressed in central nervous system (CNS) stem cells, embryonic stem cells, and some tumor cell lines (such as prostate cancer cell lines) [28–31]. GNL3 is highly expressed in the nuclei of stem cells, especially in the nucleoli. However, its expression was significantly low in nonstem cells after differentiation. Researchers found that GNL3 may regulate the proliferation cycle of stem cells and tumor cells by regulating the activity of p53. However, some researchers believe that the function of GNL3 has nothing to do with the p53 pathway in some cells [32, 33].

In this study, 20 HCC samples and their paired paracancerous tissues were detected by qRT-PCR. At the mRNA level, GNL3 expression was significantly higher in HCC than in the control group. These results suggest that GNL3 may serve as a tumor marker for the discovery of liver cancer. GEPIA database analysis showed that the high expression of GNL3 was closely related to the poor prognosis of liver cancer, which has important reference value for predicting the prognosis of liver cancer. This study also investigated the role of GNL3 in the malignant biological behavior of hepatocellular carcinoma cells. By silencing GNL3 expression in hepatocellular carcinoma cell lines, CCK-8 and Transwell assays were used to detect GNL3 proliferation and motor invasion ability. The results showed that GNL3 silencing could promote the proliferation and decrease the motor invasion of HCC cells. These results suggest that GNL3 plays a role in the malignant biology of liver cancer proliferation and motor invasion. However, the mechanism by which GNL3 regulates the biological behavior of malignant liver cancer remains unclear. This study found that GNL3 regulates the growth of HCC cells by regulating SIRT1 transcription.

Epithelial-mesenchymal transformation (EMT) refers to the transition from epithelial cells to migratory mesenchymal cells [34]. EMT plays an important pathological role in tissue fibrosis, chronic inflammation, embryo development, and tumor metastasis [35]. Tumor cells escape the anticancer barrier by enhancing cell stem cell-like activity, leading to an increase in the number of metastatic tumor cells [36]. Approximately 90% of the deaths of tumor patients are due to tumor invasion and metastasis, indicating that regulation of EMT and cell dry processes is of great significance for tumor prevention and treatment. In this study, the correlation between GNL3 and Nanog, ALDH1 and Oct4 was analysed in vitro. GNL3 maintains the stem-like properties of HCC cells by upregulating the expression of Nanog, ALDH1, and Oct4. Further validation experiments and how to regulate the molecular mechanisms related to cell stem cell-like cells need further research.

SIRT1 is responsible for maintaining the self-renewal and tumorigenicity of HCC stem cells [37]. Overexpression of exogenous SIRT1 can restore the self-renewal of nonstem cells in the liver [38]. Therefore, SIRT1 may be a molecular target for liver cancer therapy. High SIRT1 expression was correlated with tumor size, p53 expression, AFP level, and TNM stage. The overexpression of SIRT1 was positively correlated with the upregulation of PGC-1*α*. SIRT1 physiologically interacts with PGC-1*α* to deacetylate and activate PGC-1*α*. Induced PGC-1*α* increases mitochondrial copy number and mass, cellular ATP level, DNA transcription level, and mitochondrial biogenesis, promoting the migration and invasion of HCC [39, 40]. This study found that GNL3 upregulated SIRT1 expression. GNL3 mediates the stem cell-like properties of HCC cells through SIRT1. The results of this study suggest that the regulation of GNL3 and SIRT1 may be a targeted therapy to improve the malignant evolution of HCC.

This study only studied the interaction between GNL3 and SIRT1. Perhaps there are other interacting proteins in GNL3 that need further study. In addition, whether there are other interacting components of this complex in addition to GNL3/SIRT1 also needs to be explored. Why GNL3 is highly expressed in liver cancer and the mechanism has not been explained.

## 5. Conclusion

In conclusion, the high expression of GNL3 in HCC is closely related to the poor prognosis of HCC. The increased expression of GNL3 can promote the proliferation, invasion, and metastasis of HCC cells and is positively correlated with the dry cell phenotype. This study has guiding significance for the diagnosis and prognosis of hepatocellular carcinoma.

## Data Availability

The analysed data sets generated during the study are available from the corresponding author on reasonable request.
